# Synchronous bilateral primary ovarian cancer with right endometroid carcinoma and left high-grade serous carcinoma: a case report and literature review

**DOI:** 10.1186/s12905-022-01684-3

**Published:** 2022-04-05

**Authors:** Mimori Fujimori, Toshifumi Takahashi, Yuka Furukawa, Atsuhiro Takanashi, Yoshiyuki Iizawa, Masatoshi Jimbo, Shu Soeda, Keiya Fujimori, Kazuyuki Takeichi

**Affiliations:** 1 Department of Obstetrics and Gynecology, Aidu Chuo Hospital, Fukushima, Japan; 2 Fukushima Medical Center for Children and Women, Fukushima Medical University, Fukushima, Fukushima 960-1295 Japan; 3 Department of Obstetrics and Gynecology, Fukushima Medical University School of Medicine, Fukushima, Japan

**Keywords:** Ovarian cancer, Synchronous cancer, Bilateral ovarian cancer, Endometrioid carcinoma, High-grade serous carcinoma

## Abstract

**Background:**

Epithelial ovarian cancer is the most frequent gynecologic malignancy; it has a poor prognosis and often occurs bilaterally. Most cases of synchronous bilateral ovarian cancer (SBOC) are metastases from the other ovary, while bilateral primary ovarian cancer is rare.

**Case presentation:**

The patient was a 47-year-old Japanese woman with a complaint of abdominal pain for 1 month. Imaging results revealed bilateral ovarian tumors with suspicion of malignancy. The patient underwent a laparotomy with total hysterectomy, bilateral salpingo-oophorectomy, partial omentectomy, and resection of suspected dissemination in the peritoneum. Histopathological and immunohistochemical studies showed that the right ovarian tumor was an endometrioid carcinoma (G2) and had no association with endometriotic lesions. However, the left ovarian tumor was a high-grade serous carcinoma (HGSC). The final staging was stage 1 right endometrioid carcinoma and stage IIb left HGSC. Six courses of adjuvant chemotherapy with paclitaxel, docetaxel, and carboplatin were administered. The patient showed no signs of recurrence 24 months postoperatively.

**Conclusions:**

To the best of our knowledge, the combination of histological types in this case may be the first report of primary bilateral ovarian cancer. In SBOC, it is important to differentiate the subtypes of histology using immunostaining, in addition to morphopathology.

## Background

Epithelial ovarian cancer (EOC) is the most frequent malignant ovarian tumor and has the poorest prognosis among gynecological cancers [1]. There are currently no effective screening programs for EOC, and it is often found as an advanced cancer with intraperitoneal dissemination [1].

Approximately 20–25% of all cases of EOC are bilateral at the time of diagnosis [2, 3]. There are two types of synchronous bilateral ovarian cancer (SBOC): metastasis of unilateral ovarian cancer to the contralateral ovary and bilateral primary ovarian cancers. In addition, SBOCs might have the same or different histological types in each ovary. Most SBOCs have the same histology, and there are limited reports of SBOCs with different histological types in each ovary [4–11]. Studies of genetic mutations in SBOCs with the same histological type have revealed that most of them are metastatic [12, 13]. However, SBOCs with different histological types have not been adequately investigated with respect to the genetic mutations, and there is no consensus on their clinical characteristics and treatment.

We report a rare case of SBOC in which the right ovarian cancer was an endometrioid carcinoma, and the left ovarian cancer was a high-grade serous carcinoma (HGSC). To the best of our knowledge, the combination of histological types in this case may be the first report of primary bilateral ovarian cancer.

## Case presentation

**Fig. 1:**
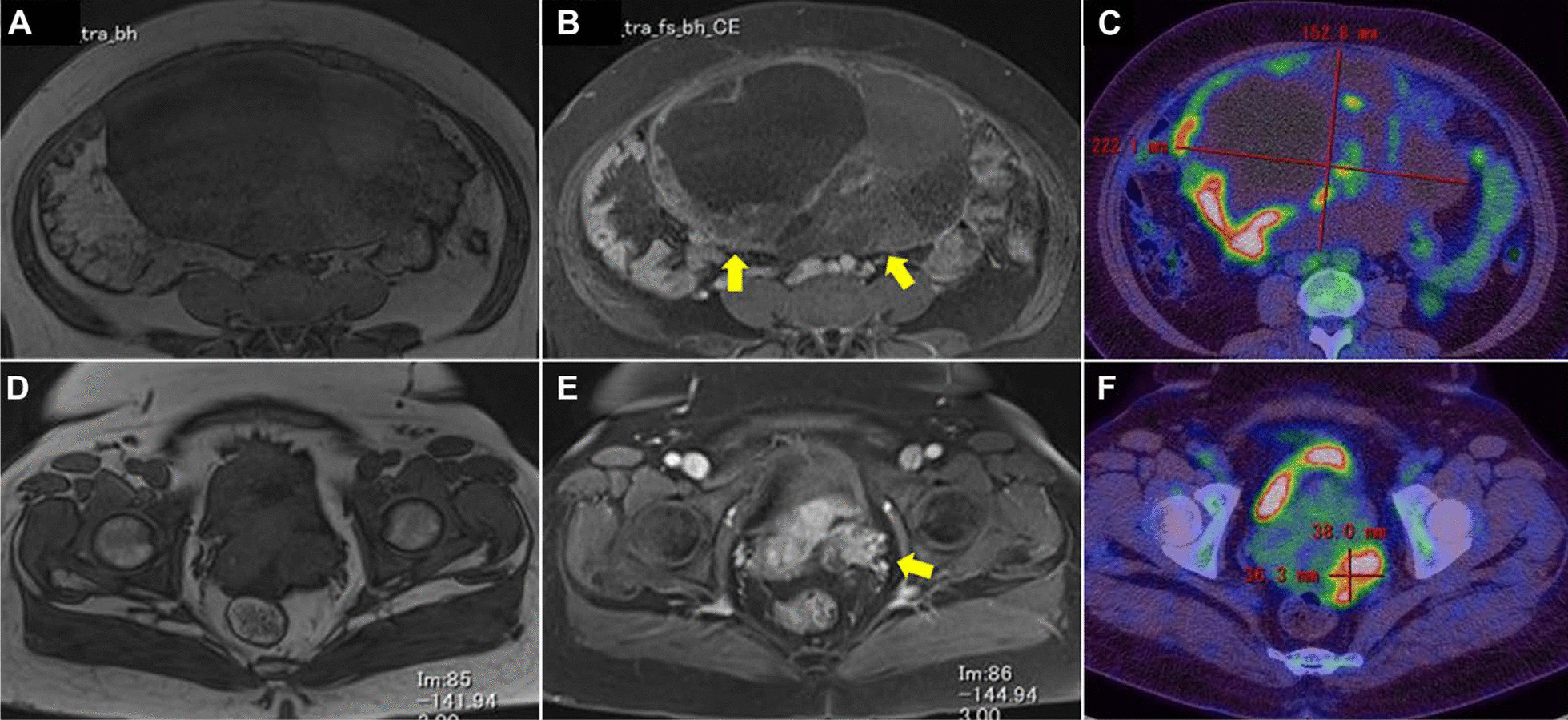
Images of MRI and PET-CT in the patient with synchronous bilateral ovarian tumor. Images of T1-weighted and T1-weighted gadolinium contrast enhancement in the right (**A**, **B**) and left (**D**, **E**) ovarian tumors. Images of PET-CT of the right (**C**) and left (**F**) ovarian tumors. Yellow arrows indicate contrast enhancement by gadolinium in the right and left ovarian tumor. *MRI* magnetic resonance imaging, *PET-CT* Positron-emission tomography and computed tomography

**Fig. 2:**
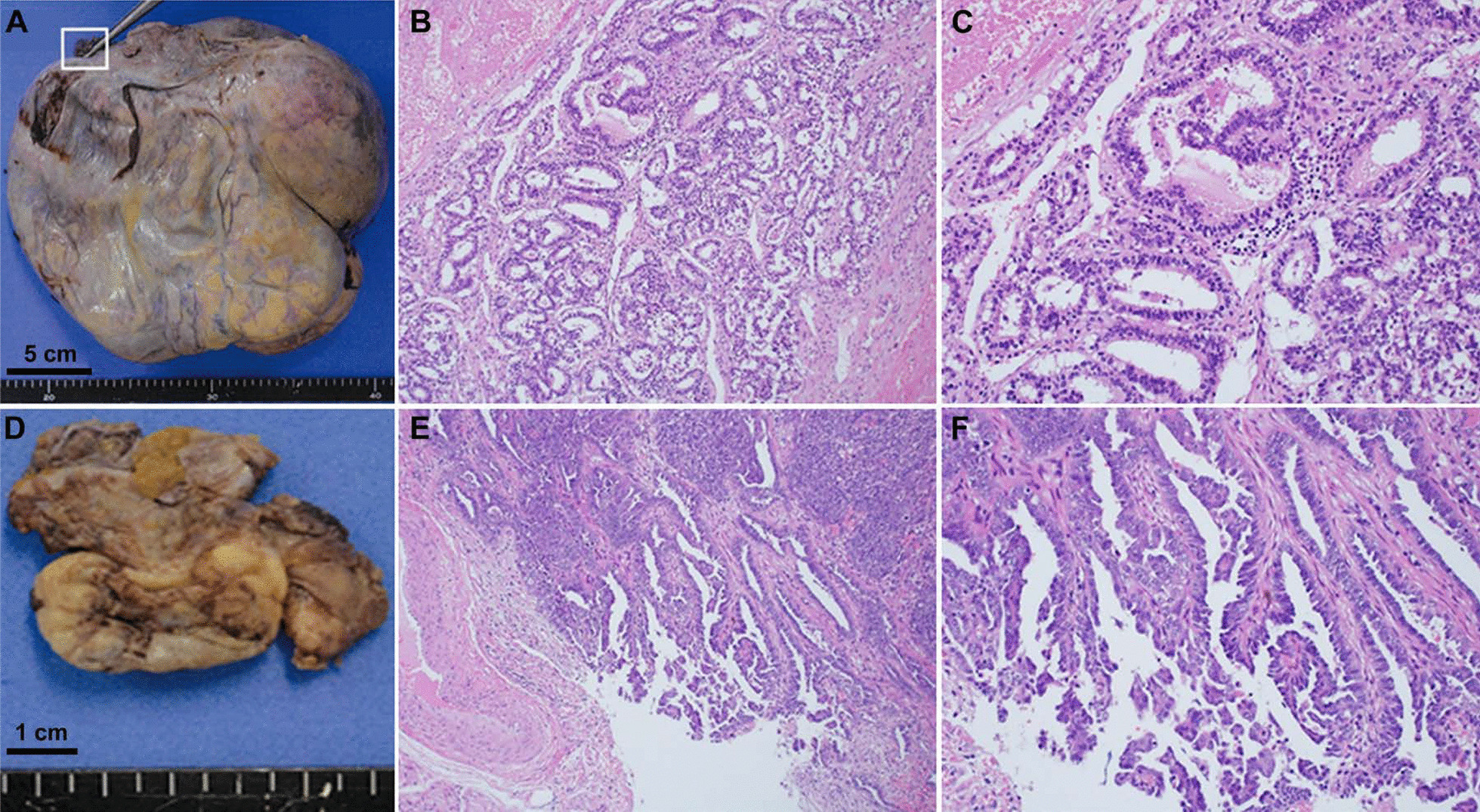
Photographs of histopathological findings of the synchronous bilateral ovarian cancers. Macroscopic specimens of the right (**A**) and left (**D**) ovarian cancers. Microscopic histopathological findings right (**B**, low magnification field; **C**, high magnification field) and left (**E**, low magnification field; **F**, high magnification field) ovarian cancers

**Fig. 3:**
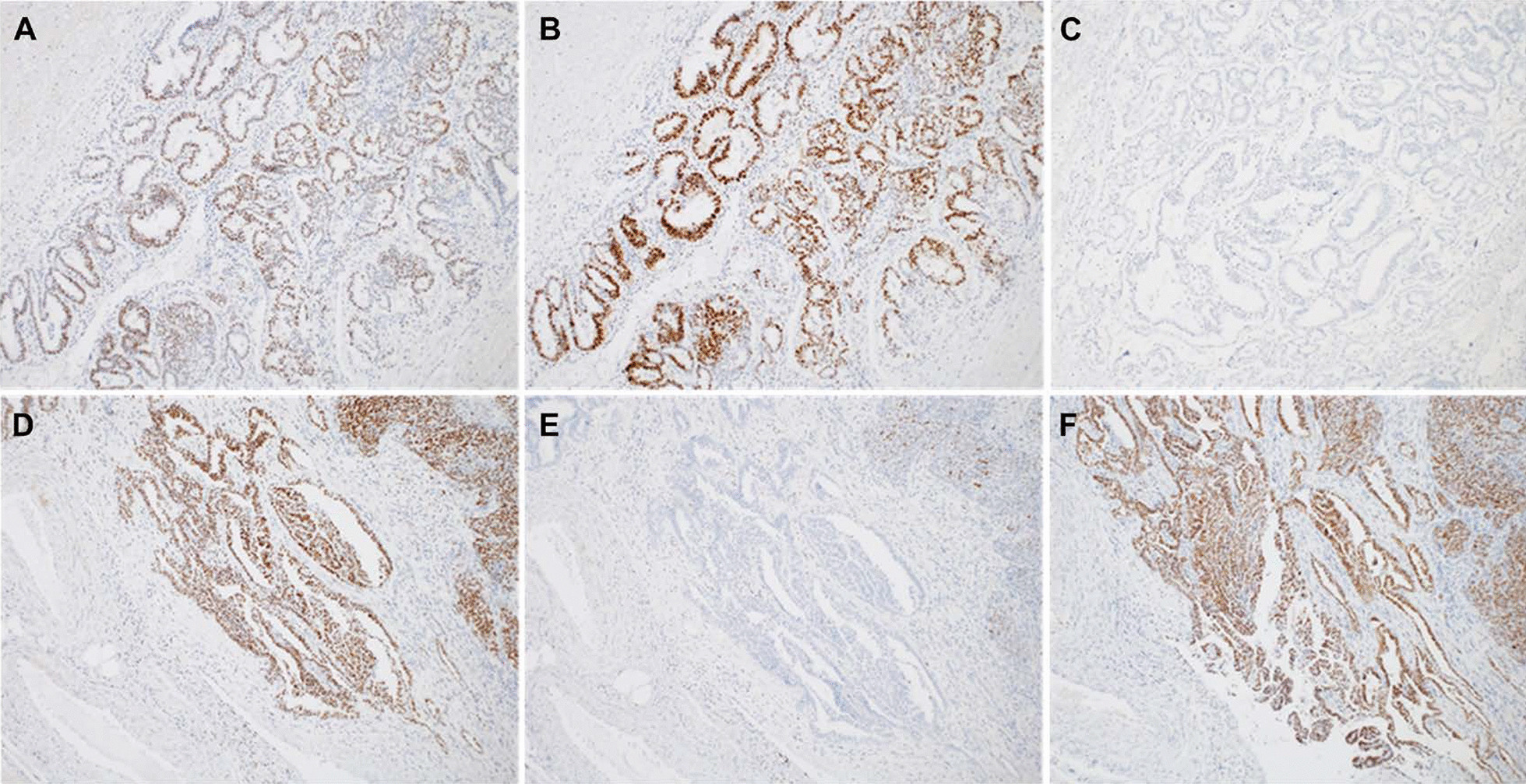
Photographs of immunohistopathological findings in the synchronous bilateral ovarian cancers. Immunodepressions of ER, PR, and WT-1 in the right (**A**–**C**) and left (**D**–**F**) ovarian cancers. *ER* estrogen receptor-alpha, *PR* progesterone receptor, *WT-1* Wilms’ tumor-1

The patient was a 47-year-old Japanese woman with no history of pregnancy or marriage. Her menarche was at the age of 11 years. Her menstrual cycle was regular, 28 days in duration, with no evidence of menorrhagia or dysmenorrhea. There was no painful defecation or dyspareunia. Her medical history included hypertension and type 2 diabetes. The family history revealed that her grandmother had breast cancer. She visited a clinic for lower abdominal pain for approximately 1 month. The patient was referred to our hospital because of suspicion of an ovarian tumor. Her height was 162 cm, and she weighed 99 kg, and her body mass index was 37.7 kg/m^2^. The abdomen was distended, and a firm mass was palpated in the right lower abdomen. There was no tenderness in the lower abdomen. Vaginal examination showed portio erosion, and the secretion was white and moderate in volume. On internal examination, there was no induration of the Douglas fossa or pain on palpation of the portio cervix. Transabdominal and transvaginal ultrasonography showed a multifocal mass with mixed cystic and solid components measuring more than 20 cm in size in the right adnexa. Cervical cytology was negative for intraepithelial neoplasia or malignancy. Hematological examination revealed no abnormal findings. The levels of tumor markers, serum CA125, carbohydrate antigen 19–9, and carcinoembryonic antigen were 228.8 U/mL (reference < 35 U/mL), 93.6 IU/mL (reference < 37 IU/mL), and 0.8 ng/mL (reference < 5.0 IU/mL), respectively. Magnetic resonance imaging revealed that the mass in the right adnexa originated from the right ovary; it was a mixed cystic and solid mass with contrast enhancement by gadolinium. The mass in the left adnexa originated in the left ovary, and similar to the right-sided mass, it was a mixed cystic and solid mass with contrast enhancement by gadolinium. Positron-emission tomography and computed tomography scan showed high 18F-fluorodeoxyglucose accumulation with maximum standardized uptake value (SUV max) of 16.2 in the right adnexal mass and SUV max of 11.8 in the left adnexal mass (Fig. 1). Since the diagnosis was bilateral ovarian tumor, upper gastrointestinal endoscopy was performed to differentiate the metastatic ovarian tumor; however, there were no abnormal findings. In addition, there were no palpable masses in both breasts. The patient was suspected to have ovarian cancer, and radical surgery via laparotomy was performed. On laparotomy, there were no adhesions or endometriotic lesions in the abdominal cavity. A small amount of ascitic fluid was found in the Douglas fossa. Bilateral ovarian tumors were observed, and the right ovarian tumor was adherent to the retroperitoneum but could be dissected manually. A simple total hysterectomy, bilateral salpingo-oophorectomy, and partial omentectomy were performed. In addition, suspected dissemination to the rectal surface and pelvic peritoneum was resected. Macroscopic findings showed a right ovarian tumor measuring 22 × 19 cm and a left ovarian tumor measuring 3.0 × 1.7 cm. Histopathological examination of the excised specimens showed that the right ovarian tumor was a solid mass with cystic changes, and the histological type was endometrioid carcinoma (G2) with no association with endometriotic lesions. The left ovarian tumor was a papillary and solid mass with histological findings of HGSC (Fig. 2). There was dissemination of HGSC in the pelvic peritoneum. There were no neoplastic changes in both fallopian tubes and no metastases to the omentum, uterine endometrium, or cervix. Immunohistochemical staining was performed using antibodies against Wilms’ tumor-1 (WT-1), estrogen receptor (ER), progesterone receptor (PR), and p53, to differentiate between the histological types of the right and left ovarian tumors. The results showed that the right ovarian tumor, an endometrioid carcinoma, was negative for WT-1, positive for ER and PR, negative for p53, and negative for napsin-A, which is expressed in clear cell carcinoma. The left ovarian HGSC was WT-1 positive, ER-positive, PR-negative, and p53-negative (Fig. 3). Histopathological and immunohistological examinations revealed the simultaneous presence of right endometrioid adenocarcinoma and left HGSC, independent of each other. The final staging was stage 1 right endometrioid carcinoma and stage IIb left HGSC (TNM classification, pT2bNxM0). Adjuvant chemotherapy with paclitaxel (330 mg) and carboplatin (700 mg of the area under the curve = 4) was administered. However, grade-3 allergic symptoms, such as generalized urticaria, appeared after one course, and the regimen was changed to docetaxel and carboplatin for a total of six courses. The patient showed no signs of recurrence 24 months postoperatively.

## Discussion and conclusions

We encountered a rare case of SBOC with different histological types, namely, endometrial carcinoma in the right ovary and HGSC in the left ovary. To the best of our knowledge, the combination of histological types in this case may be the first report of primary bilateral ovarian cancer.

Synchronous occurrence of primary cancers of the genital tract is rare, and the incidence is reported to be approximately 1–2% [14–17]. The most common combination of genital cancers with synchronous occurrence in different organs is ovarian and uterine cancers. Even in early-stage ovarian cancer, it is found simultaneously in the left and right ovaries in approximately 20–25% of the cases at the time of diagnosis [2, 3]. Most SBOCs are considered to be metastases from unilateral ovarian cancer. Yin et al. analyzed 12 SBOCs (11 HGSCs and 1 endometrioid carcinoma) by whole genome sequencing and performed a genetic analysis of the left and right ovarian cancers [12]. They reported that the bilateral ovarian cancers appeared to have originated from similar clones. In addition, Li et al. also analyzed 13 SBOCs (11 serous carcinomas and 2 clear cell carcinomas) using whole-exome sequencing and found that they had an average of 68.5% common somatic mutations. Considered together, it is reasonable to conclude that SBOC with the same histology is an early metastasis due to intraperitoneal dissemination. However, a comprehensive analysis of gene mutations in SBOC with histological differences has not been performed because of their rarity. The study of gene mutations in SBOC with different histological types is important for the further understanding of ovarian cancer.

If the histological diagnosis of the left and right ovarian cancers is different, it is considered as simultaneous primary ovarian cancer. Table 1 summarizes the reported cases of SBOC with different histological types in each ovary, including the eight previously reported cases and our case [4–11]. Combinations of different histological types have been reported, including 4 cases of endometrioid carcinoma and clear cell carcinoma, 2 cases of malignant mixed Müllerian tumor (MMMT) and serous carcinoma, 2 cases of HGSC and clear cell carcinoma, and our case of endometrioid and HGSC. The combination of endometrioid carcinoma and HGSC seen in this case has not been reported previously.

**Table 1:** Reported cases of synchronous bilateral ovarian cancer with different histopathology in each ovary

First author (year)	Patient age	Type of histopathology of right ovarian cancer	Type of histopathology of left ovarian cancer	Presence of endometriosis in the tumors	Immunohistochemical study	Staging of ovarian cancer*	Types of surgery	Types of adjuvant chemotherapy	Prognosis
Song (2011) [4]	58	Malignant mixed Müllerian tumor (MMMT)	Serous papillary adenocarcinoma	None in both tumors	Rt: CK^a^ + , vimentin+, S-100+, actin−, desmin−, CD34^−^ Lt: NA	IIIB	TAH^j^ + BSO^k^ + PLA^l^ + PAN^m^ + OME^n^	TC^o^ for 5 cycles	Recurrence and DOD 7 months after the primary treatment
Bernardez Zapata (2012) [5]	49	Endometrioid carcinoma (G2)	Clear cell carcinoma	Yes, in the right ovarian tumor	Rt: WT^i^-1+, ER^f^+, PR^h^+ Lt: CK7^b^+, p53+,	IB	TAH + BSO + PLA + OME	NA	NA^r^
Nayal (2014) [7]	38	High-grade serous papillary adenocarcinoma	Clear cell carcinoma	None in both tumors	NA	IIB	TAH + BSO + OME	TC for 6 cycles	NED^q^ at 2 years after the treatment
Preeti (2014) [8]	60	High-grade serous carcinoma	Clear cell carcinoma	None in both tumors	Rt: CK7+, CEA+, CD15+, WT-1+ Lt: CK7+, WT-1, ER+, p53+, CK20^c^-, CEA^d^-, CD15-	IB	TAH + BSO + OME	Chemotherapy with unknown details	NED at 4 months after the surgery
Khandeparkar (2014) [6]	65	Clear cell carcinoma	Endometrioid carcinoma	Yes, in the left ovarian tumor	Rt: p53+, ER+, WT-1-, Lt: ER+, PR+, p53+, EMA^e^+	IB	TAH + BSO + OME + appendectomy + peritoneal biopsy	NA	NA
Gunakan (2018) [9]	38	Malignant mixed Müllerian tumor	Serous carcinoma (G3)	None in both tumors	NA	IIIA1	TAH + BSO + PLA	TC 6 cycles	Recurrence and DOD^s^ after 25 months after the initial diagnosis
Zhao (2108) [10]	51	Endometrioid carcinoma	Clear cell carcinoma	Yes, in the left ovarian tumor	Rt: NA Lt: CD15+, CK7+, CK20−, Napsin-A+, p53 + ER + , PR +	IC2	RSO (prior surgery) and TAH + LSO + PLA + PAN + OME + appendectomy	TC for 5 cycles	NED at 1 year after the treatment
Sao (2020) [11]	60	Endometrioid carcinoma (G1)	Clear cell carcinoma	Yes, in both tumors	Rt: ER + , Napsin-A- Lt HNF^g^-1β + , Napsin-A + , ER-	IIA	TAH + BSO + PLA + PAN + OME + appendectomy	TC	NA
Fujimori (present case)	47	Endometrioid carcinoma (G2)	High-grade serous carcinoma	None in both tumors	Rt: ER + , PR + , WT-1-, p53-, Napsin-A- Lt: ER + , WT-1 + , PR-, p53-	IIB	TAH + BSO + OME + peritoneal biopsy	TC/DC^p^ for 6 cycles	NED at 1 year after the surgery

^a^*CK* cytokeratin, ^b^*CK7* cytokeratin 7, ^c^*CK20* cytokeratin 20, ^d^*CEA* carcinoembryonic antigen, ^e^*EMA* epithelial membrane antigen, ^f^*ER* estrogen receptor, ^g^*HNF-1β* hepatocyte nuclear factor-1β, ^h^*PR* progesterone receptor, ^i^*WT-1* Wilms’ tumor-1, ^j^*TAH* total abdominal hysterectomy, ^k^*BSO* bilateral salpingo-oophorectomy, ^l^*PLA* pelvic lymphadenectomy, ^m^*PAN* para-aortic lymphadenectomy, ^n^*OME* omentectomy, ^o^*TC* paclitaxel and carboplatin, ^p^*DC* docetaxel and carboplatin, ^q^*NED* no evidence of disease, ^r^*NA* not available, ^s^*DOD* death due to disease

Endometrioid carcinoma accounts for 15–19% of all EOCs [18]. Some endometrioid carcinomas originate from endometriotic lesions [19], and nearly 50% of all endometrioid carcinomas have been reported to be associated with endometriotic lesions in the ipsilateral ovary or pelvic peritoneum [20]. In SBOC with different histology in both ovaries, the four cases of endometrioid and clear cell carcinoma were associated with endometriosis (Table 1) [5, 6, 10, 11]. However, in our case, there were no endometriotic lesions in the peritoneum and ovary.

Serous carcinoma is the most frequent histological type in all EOCs [18]. Serous carcinoma is classified into low- and high-grade carcinomas, and most of them are HGSC. HGSC is oviductal in origin, and 50–60% of HGSCs have serous tubal intraepithelial carcinoma (STIC) [21]. Three cases of SBOCs were found to have a serous carcinoma in one ovary; in two of these cases, they were associated with clear cell carcinoma (Table 1) [4, 7, 8]. In these three cases, there is no information about the presence or absence of STIC. In our case, STIC lesions were not observed in the fallopian tube.

When SBOC is encountered, immunostaining for tissue-specific protein expression, in addition to histopathological diagnosis, is useful in differentiating the ovarian cancer subtypes. Endometrioid carcinoma is usually positive for cytokeratin 7 (CK7), CA125, ER, PR, PAX8, and hepatocyte nuclear factor 1-β, while HGSC is positive for CK7, CA125, WT-1, p16, and PAX8 [22]. In particular, WT-1 is almost always negative in endometrioid carcinoma, while it is positive in HGSC and is useful for differentiation. However, it is sometimes difficult to distinguish poorly differentiated endometrioid carcinoma from HGSC, and it has been reported that WT-1 is positive in poorly differentiated endometrioid carcinoma [23]. p53 mutations are found in 95% of HGSC carcinomas, which are detected by immunohistochemistry in only 60% of the cases [24]. Clear cell carcinoma is the second most common type of ovarian cancer after serous carcinoma in Japan. It has been reported that napsin-A is frequently expressed in clear cell carcinoma [25] and is often associated with endometriosis. The detection of napsin-A is useful in differentiating endometriosis-related cancers, such as endometrioid carcinoma and clear cell carcinoma. In our case, immunostaining for WT-1, ER, PR, p53, and napsin-A revealed that ER and PR were positive and WT-1, p53, and napsin-A were negative in endometrioid carcinoma, while WT-1 and ER were positive and PR and p53 were negative in HGSC.

The standard treatment for SBOC has not yet been established. In the case series of SBOC with different histology, the standard treatment for ovarian cancer, such as adjuvant chemotherapy, was administered after staging surgery in all cases as an initial treatment (Table 1) [4–11]. The two cases with MMMT and serous carcinoma were stage III and had a poor prognosis, as the patients developed recurrence and died after surgery and adjuvant chemotherapy [4, 9]. In these cases, the recurrence was of the MMMT ovarian cancer. As in our case, the treatment strategies for endometrioid carcinoma and HGSC are essentially the same. For advanced-stage ovarian cancer, postoperative adjuvant chemotherapy with paclitaxel and carboplatin (TC) is recommended in addition to radical surgery. In our case, mixed TC and docetaxel carboplatin therapies were administered due to grade-3 allergic symptoms caused by the TC regimen.

In conclusion, this was a rare case of SBOC with endometrioid carcinoma in the right ovary and HGSC in the left ovary. In SBOC, it is important to differentiate the histologic subtypes using immunostaining, in addition to morphopathology. Since standard treatment has not been established for synchronous bilateral primary ovarian cancer, a strict and close follow-up is necessary to detect the recurrence of cancer.

## Data Availability

Not applicable.

## Abbreviations

EOC: Epithelial ovarian cancer
SBOC: Synchronous bilateral ovarian cancer
HGSC: High-grade serous carcinoma
SUV max: Standardized uptake value
ER: Estrogen receptor
PR: Progesterone receptor
MMMT: Malignant mixed Müllerian tumor
STIC: Serous tubal intraepithelial carcinoma
TC: Paclitaxel and carboplatin

## References

[1] Lheureux S, Gourley C, Vergote I, Oza AM (2019). Epithelial ovarian cancer. Lancet.

[2] Park TW, Felix JC, Wright TC (1995). X chromosome inactivation and microsatellite instability in early and advanced bilateral ovarian carcinomas. Cancer Res.

[3] Micci F, Haugom L, Ahlquist T, Abeler VM, Trope CG, Lothe RA (2010). Tumor spreading to the contralateral ovary in bilateral ovarian carcinoma is a late event in clonal evolution. J Oncol.

[4] Song MJ, Lee CW, Seo KJ, Kim JA, Park JS, Hur SY (2011). A case of bilateral ovarian synchronous tumors (left ovarian serous papillary adenocarcinoma and right ovarian malignant mixed Mullerian tumor). Eur J Gynaecol Oncol.

[5] Bernardez Zapata F, Jauregui Melendrez RA, Cabrera CE (2012). Synchronous double primary ovarian tumor in situ. Ginecol Obstet Mex.

[6] Khandeparkar SG, Deshmukh SD, Lekawale HS, Bhoge A, Ahmed AT (2014). A rare case of synchronous right ovarian clear cell carcinoma and an incidental left ovarian endometrioid carcinoma with immunohistochemical study. J Midlife Health.

[7] Nayal B, Mathew M, Rao L, Nagel B, Kumar P (2014). Synchronous bilateral clear cell carcinoma and papillary serous cystadenocarcinoma of the ovaries. J Interdiscip Histopathol.

[8] Preeti A, Arunachalam KA, Pradeep Y, Mati GM (2014). Bilateral synchronous high-grade serous carcinoma and clear cell carcinoma in right and left ovaries with immunohistochemical confirmation: an exceptional finding. Indian J Pathol Microbiol.

[9] Gunakan E, Tohma YA, Haberal AN, Ayhan A (2018). Bilateral synchronous ovarian tumours: an uncommon case and review of the literature. Prz Menopauzalny.

[10] Zhao LJ, Wang P, He Y (2018). Synchronous occurrence of primary right ovarian endometrioid adenocarcinoma and primary left ovarian clear cell adenocarcinoma: a case report. Medicine (Baltimore).

[11] Sao CH, Lai WA, Lin SC, Chang CM, Chen YJ, Wang PH (2020). Endometriosis-associated epithelial ovarian cancer: primary synchronous different cellular type on each ovary. Taiwan J Obstet Gynecol.

[12] Yin X, Jing Y, Cai MC, Ma P, Zhang Y, Xu C (2017). Clonality, heterogeneity, and evolution of synchronous bilateral ovarian cancer. Cancer Res.

[13] Li C, Bonazzoli E, Bellone S, Choi J, Dong W, Menderes G (2019). Mutational landscape of primary, metastatic, and recurrent ovarian cancer reveals c-MYC gains as potential target for BET inhibitors. Proc Natl Acad Sci U S A.

[14] Eser S, Gulhan I, Ozdemir R, Dicle N, Hanhan M, Baloglu A (2011). Synchronous primary cancers of the female reproductive tract in Turkish women. Asian Pac J Cancer Prev.

[15] Ayhan A, Yalcin OT, Tuncer ZS, Gurgan T, Kucukali T (1992). Synchronous primary malignancies of the female genital tract. Eur J Obstet Gynecol Reprod Biol.

[16] Tong SY, Lee YS, Park JS, Bae SN, Lee JM, Namkoong SE (2008). Clinical analysis of synchronous primary neoplasms of the female reproductive tract. Eur J Obstet Gynecol Reprod Biol.

[17] Singh N (2010). Synchronous tumours of the female genital tract. Histopathology.

[18] Machida H, Matsuo K, Yamagami W, Ebina Y, Kobayashi Y, Tabata T (2019). Trends and characteristics of epithelial ovarian cancer in Japan between 2002 and 2015: a JSGO-JSOG joint study. Gynecol Oncol.

[19] Bulun SE, Wan Y, Matei D (2019). Epithelial mutations in endometriosis: link to ovarian cancer. Endocrinology.

[20] Terada T (2012). Endometrioid adenocarcinoma of the ovary arising in atypical endometriosis. Int J Clin Exp Pathol.

[21] Przybycin CG, Kurman RJ, Ronnett BM, Shih Ie M, Vang R (2010). Are all pelvic (nonuterine) serous carcinomas of tubal origin?. Am J Surg Pathol.

[22] Yasuda M, Kato T, Suzuki H (2011). Topics in ovarian tumors: ovarian tumors and immunohistochemistry. Pathol Clin Med.

[23] McCluggage WG (2008). My approach to and thoughts on the typing of ovarian carcinomas. J Clin Pathol.

[24] Kobel M, Ronnett BM, Singh N, Soslow RA, Gilks CB, McCluggage WG (2019). Interpretation of P53 immunohistochemistry in endometrial carcinomas: toward increased reproducibility. Int J Gynecol Pathol.

[25] Yamashita Y, Nagasaka T, Naiki-Ito A, Sato S, Suzuki S, Toyokuni S (2015). Napsin A is a specific marker for ovarian clear cell adenocarcinoma. Mod Pathol.

